# Shift Work and Heart Rate Variability Coherence: Pilot Study Among Nurses

**DOI:** 10.1007/s10484-018-9419-z

**Published:** 2018-09-19

**Authors:** James B. Burch, Melannie Alexander, Pallavi Balte, Jameson Sofge, James Winstead, Venkat Kothandaraman, J. P. Ginsberg

**Affiliations:** 1 Department of Epidemiology and Biostatistics, Arnold School of Public Health, University of South Carolina, Columbia, SC USA; 2 WJB Dorn Department of Veterans Affairs Medical Center, Columbia, SC USA; 3 Columbia University, New York, NY USA; 4 Department of Biological Sciences, University of South Carolina, Columbia, SC USA; 5 Department of Pharmacology, Physiology and Neuroscience, School of Medicine, University of South Carolina, Columbia, SC USA; 6 Department of Epidemiology and Biostatistics, Arnold School of Public Health, University of South Carolina, 915 Greene Street, Room 226, Columbia, SC 29208 USA

**Keywords:** Autonomic, Cardiac coherence, Circadian, Night shift, Parasympathetic, Sleep

## Abstract

This study used ambient heart rate monitoring among health care workers to determine whether a novel measure of heart rate variability (HRV), as well as sleep disturbances, fatigue, or cognitive performance differed among non-rotating night shift nurses relative to those working permanent day shifts. Continuous ambulatory HRV monitoring was performed among night nurses (n = 11), and a comparison group of permanent day nurses (n = 7), over a 36-h period coinciding with the last two 12-h shifts of each participant’s work week. Symptoms and psychomotor vigilance were assessed at the end of the ambient HRV monitoring period, and no differences between shifts were observed. Day nurses exhibited an increase in hourly mean HRV coherence ratios during their sleep period, suggesting a circadian pattern of cardiorespiratory phase coupling, whereas night nurses had no increase in HRV coherence ratios during their sleep period. The HRV coherence patterns were similar to high frequency HRV power among nurses on the same shift. To the authors knowledge, this study was the first to quantify patterns of the HRV coherence ratio among shiftworkers in a non-experimental (work/home) setting. The results suggest a pattern of autonomic dysregulation among night workers during their sleep period relative to those working day shifts. The HRV coherence ratio may serve as a novel indicator of HRV dysregulation among shift workers.

## Introduction

Shift work is an essential component of modern clinical workplaces, and it has been associated with assorted maladies, including: sleep disruption, fatigue, cognitive impairment, accidents, injuries, depression, metabolic and gastrointestinal disturbances, and increased risks for diabetes, cardiovascular disease, and cancer (Reutrakul and Knutson 2015; Videnovic and Zee 2015; IARC 2010; Faraut et al. 2013; Folkard et al. 2005; Caruso 2014). About 20–30% of US employees work some type of irregular shift, and the work includes high-consequence occupations, including: health care, transportation, public safety, and emergency response (McMenamin 2007; Yong et al. 2017). Understanding how to ameliorate the pathophysiological perturbations induced by shift work is essential for implementation of effective strategies for safety and disease prevention. Shift work and its accompanying disruption of circadian rhythms and sleep act as stressors that can activate the sympathetic nervous system, increase allostatic load, and stimulate inflammatory cytokine secretion (Caruso 2014; Vogel et al. 2012; Puttonen et al. 2011; Irwin et al. 2016; Dettoni et al. 2012). These perturbations occur in conjunction with neuroendocrine and lipid dysregulation, hypertension, and other cardiometabolic disturbances that are observed among shift workers (Reutrakul and Knutson 2015; Proper et al. 2016).

A normally functioning cardiovascular system depends on a homeostatic balance between the sympathetic and parasympathetic components of the autonomic nervous system (ANS). The ANS interacts closely with the cardiopulmonary system, and the activity of both systems exhibit distinct circadian patterns that are readily quantified via monitoring of blood pressure or heart rate (Takeda and Maemura 2016; Portaluppi et al. 2012). Under normal circumstances, average heart rate increases in the morning after awakening, reaches a peak between 1000 and 1200 h, then gradually declines later in the day, maintaining a low level during the night relative to daytime (Takeda and Maemura 2016; Portaluppi et al. 2012). Adverse cardiovascular events, including myocardial infarction, sudden cardiac death, pulmonary embolism, and ischemic or hemorrhagic stroke, all exhibit a circadian pattern of risk, with a greater likelihood of occurrence in the early morning relative to night (Takeda and Maemura 2016; Manfredini et al. 2005; Portaluppi et al. 2012). These observations emphasize the importance of circadian processes in cardiovascular pathology. Susceptibility to desynchronization of circadian autonomic and cardiopulmonary processes may influence one’s ability to adapt to shift work. In one study, night workers who were physiologically adapted to their schedule had better performance, alertness, and mood, as well as longer daytime sleep and lower sympathetic ANS output during their sleep period, relative to non-adapted night workers (Boudreau et al. 2013). It is logical to assume that maladapted shift workers are at greater risk of developing adverse health outcomes relative to adapted workers, although this possibility has not been thoroughly investigated.

The interplay between the ANS and cardiopulmonary systems can be readily monitored via quantification of heart rate variability (HRV), which has been used as a health indicator in numerous clinical settings. Decreased HRV has been associated with: work-related stress, anxiety, depression, increased secretion of pro-inflammatory mediators, cardiovascular disease risk factors, as well as increased risks for myocardial infarction, stroke, and mortality, particularly among those with a pre-existing chronic disease (Aeschbacher et al. 2017; Zhou et al. 2016; Thayer et al. 2010; Tsuji et al. 1996; Dekker et al. 2000). Given the association between long-term shift work and cardiovascular disease (Reutrakul and Knutson 2015; Puttonen et al. 2010; Wang et al. 2011), the influence of shift work and sleep disturbances on HRV and autonomic function has been an active area of investigation (Souza et al. 2015; Puttonen et al. 2010; Stein and Pu 2012; Jarczok et al. 2013; Togo and Takahashi 2009). HRV also has been targeted as a candidate biomarker for monitoring shift work adaptation (Boudreau et al. 2013; Chung et al. 2009, 2012). Ambulatory HRV monitoring is a valid, non-invasive procedure that can be used to examine autonomic function as it relates to the cardiopulmonary system over extended periods (e.g., continuously for up to several days), and over important circadian time periods (e.g., during sleep). However, only a limited number of studies have examined ANS activity among shift workers over extended periods (Amirian et al. 2014; Jarvelin-Pasanen et al. 2013; van Amelsvoort et al. 2001; Ito et al. 2001; Boudreau et al. 2013; Wehrens et al. 2012; Lindholm et al. 2012; Jensen et al. 2016).

Appropriate interactions between the cardiac and respiratory cycles, achieved through balanced ANS activity, are critical for homeostasis and survival. Strategies designed to increase HRV and vagal parasympathetic output have been suggested for eliciting a number of health benefits including reduced mortality risk (Dunlap et al. 2015; Wheat and Larkin 2010; Garcia et al. 2013). ‘HRV coherence’ refers to a state of physiological entrainment between HRV, respiration, and the baroreflex. When in HRV coherence, vagal parasympathetic tone increases, pulmonary gas exchange is optimized, and homeostasis between the ANS and cardiopulmonary systems is achieved (Park and Thayer 2014; Lang and Bradley 2010; Garcia et al. 2013). HRV coherence can be achieved by practicing a paced breathing exercise (resonant frequency breathing) using a procedure known as HRV biofeedback (Wheat and Larkin 2010; Gevirtz 2013; Lehrer et al. 2000; Lehrer and Gevirtz 2014). Results from interventions targeting HRV coherence suggest that it may alleviate adverse impacts encountered among shift workers by facilitating improvements in cognitive performance and sleep, and by reducing stress, anxiety, and depression (Goessl et al. 2017; Thayer et al. 2009; Lehrer 2018; Lehrer and Gevirtz 2014; Morgan and Mora 2017; Sakakibara et al. 2013). Additionally, the restorative properties of deep sleep are considered to be related to the parasympathetic dominance, resonant frequency breathing, and cardiorespiratory coupling that occur during non-rapid eye movement (NREM) and slow-wave sleep (Tobaldini et al. 2013; Cabiddu et al. 2012; Penzel et al. 2016; Jerath et al. 2014). However, to the authors’ knowledge, human HRV coherence has never been quantified in a non-experimental setting during either work or sleep periods. This study tested the hypotheses that HRV coherence and symptoms of sleep disturbance, fatigue, and psychomotor vigilance differ among night nurses relative to those working day shifts. Ambulatory heart rate monitoring was performed over a 36-h period among nurses working permanent nights, and in a comparison group working permanent day shifts. The pattern of HRV coherence was examined over the study period along with other representative HRV measures, and symptoms were assessed at the end of the ambulatory monitoring period.

## Methods

The study population was comprised of registered nurses and/or nursing staff working in the intensive care units of a regional Department of Veterans Affairs Medical Center. Nurses at least 18 years old of any race, ethnicity, or sex were eligible. Those with recent transmeridian travel or less than 1 month of work duration on their currently assigned shift were excluded (if their previous position was different from their current work schedule). Recruitment was performed by providing a verbal description of the study aims, methods, procedures and time commitment during a scheduled staff meeting. The night nurses’ schedules consisted of permanent (non-rotating) 12-h shifts from 7:00 pm to 7:00 am for 3 or 4 days per week over a 2-week period. Day nurses worked a similar non-rotating schedule from 7:00 AM to 7:00 pm. Informed consent was obtained from all participants, and the study was approved by the Institutional Review Board. Participants were studied over one 36-h period coinciding with the last two shifts of their 3- or 4-day work week (a few subjects had up to 39 h of monitoring data). Each participant wore a portable, data-logging electrocardiogram (ECG) heart rate monitor (FirstBeat Bodyguard, Jyväskylä, Finland) at all times (except for showering or bathing) that provided a continuous recording of R–R heart beat intervals starting at the beginning of one 12-h work shift and stopping at the end of the following work shift.

HRV data were binned into hourly segments and processed according to established guidelines (Camm et al. 1996). KUBIOS software was used to de-artifact raw data and perform a fast Fourier transformation of the HRV power spectrum for each data file. After de-artifacting, the HRV coherence ratio was calculated by identifying the maximum peak in the 0.04–0.26 Hz range, calculating the integral in a window 0.030 Hz wide centered on the highest peak in that region (‘peak power’, usually ~ 0.1 Hz), then calculating the total power of the entire spectrum. The coherence ratio was then formulated as: peak power/(total power–peak power). The reason for selection of the peak power in the frequency range of 0.04–0.26 Hz is because this is the range within which cardiorespiratory entrainment occurs, and it serves as an indicator of vagal tone (Lehrer et al. 2000; Ginsberg et al. 2010; McCraty and Zayas 2014; Lehrer and Gevirtz 2014). The average HRV coherence ratio was calculated for each 1-h segment. In addition, the hourly averages of time-domain [e.g., standard deviation of heart rate N–N intervals (SDNN), and the square root of the mean squared difference of successive N–N intervals (RMSSD)], and frequency-domain variables [e.g., high frequency (HF) power] also were calculated.

At the end of the final shift during which HRV monitoring occurred, participants completed questionnaires to obtain information on: medical conditions (having ever been told they had: hypertension, diabetes, elevated cholesterol, high or low thyroid levels, anemia, or any other physician diagnosed illness; use of prescribed or non-prescribed medications; or a family history of heart disease); lifestyle (physical activity, use of tobacco products, alcoholic or caffeinated beverages, multivitamins); and sociodemographics (e.g., age, race, sex, height, weight, education, income). Questionnaires were completed in a private setting and data were coded to ensure participant confidentiality. Physical activity was assessed using the Rapid Assessment of Physical Activity (RAPA) instrument (Topolski et al. 2006). Shift work-related information was obtained from the Standard Shift work Index (Barton et al. 1995). Domains of fatigue were assessed using the Multidimensional Fatigue Inventory (MFI) short form (Smets et al. 1995). This instrument is comprised of 20 questions that assess general, physical, and mental fatigue, as well as reduced motivation and activity. It has good internal consistency (Cronbach alpha: > 0.75) and high convergent validity (Smets et al. 1995, 1996; Lin et al. 2009). Scores on each subscale range from 4 to 20, with higher scores indicating greater fatigue.

Sleep disturbances were assessed using the Pittsburg Sleep Quality Index (PSQI) (Buysse et al. 1989), a self-administered 19-item instrument that includes seven components assessing sleep: quality, onset latency, duration, efficiency, and disturbance, as well as sleep medication use and daytime dysfunction (score range 0–21, with higher scores representing more sleep disruption). The Cronbach alpha coefficient for all component scores (0.83) indicated high internal consistency (Buysse et al. 1989). Those with disorders of sleep initiation or maintenance, or excessive somnolence, had higher scores compared to controls, indicating good construct validity. When used to identify patients with sleep disorders, a total PSQI score ≥ 5.0 had a sensitivity of 90% and a specificity of 87% (Buysse et al. 1989). Information from the PSQI on usual sleep and wake time was included with plots of hourly HRV coherence measures.

Following completion of the questionnaires, the psychomotor vigilance task (PVT) was used to assess sustained attention using a pre-programmed portable monitor (PVT-192, Ambulatory Monitoring, Inc., Ardsley, NY) (Dinges and Powell 1985; Khitrov et al. 2014; Basner et al. 2011). The PVT is advantageous since it is not influenced by aptitude, learning, or practice effects, and it has high test–retest reliability (Dinges et al. 1997; Basner et al. 2011). Participants were presented with a visual stimulus over 5 min and asked to press a button on the monitor with their dominant hand when they first detected the stimulus. The inter-stimulus intervals and response times (ms) were recorded, and the median reaction time and the number of response lapses (reaction time > 500 ms) were included in the analysis. Reduced response speed and a greater number of lapses are indicative of sleep deprivation and reduced performance (Tkachenko and Dinges 2018).

Statistical analyses were performed using the Statistical Analysis Software computer program (SAS version 9.4, Cary, NC). Descriptive characteristics of participants were summarized among all nurses and according to shift status. Demographic, lifestyle and psychometric variables were compared between work shifts using the Student *t* test for normally distributed continuous variables, the Wilcoxon exact test for non-normally distributed continuous variables, or Fisher’s exact test of independence for discrete variables. Continuous outcomes and cumulative years of shift work were evaluated as dichotomous variables using the median among all participants as the cutpoint (12 years for cumulative shift work). Psychometric characteristics of sleep, fatigue, and performance (PVT) were compared between day and night shift workers using multivariable linear regression, after adjusting for demographic or lifestyle variables that differed between shifts (p < 0.05).

Hourly average HRV data were organized from the beginning to the end of the 36-h monitoring period (shift hours 1–36, respectively) and displayed graphically by work shift. A mixed effects, repeated measures model (SAS ‘Proc Mixed’, alpha: 0.05, two-tailed) was used to evaluate changes in mean HRV coherence ratios across the 36-h recording period, between shifts, and for the shift by hour interaction. Pre-planned contrasts at each time point compared hourly averages of the HRV Coherence Ratio among nurses working nights relative to those working days using independent *t* tests at each shift hour and p-values adjusted to protect against inflation of the familywise Type I error rate (McDonald 2014; Toothacker 1993). Hochberg’s step-up method for multiple testing was implemented using a false discovery rate of 0.05, and 36 h as the ‘family of tests’ (Hochberg 1988). In addition, the pattern of 36-h HRV coherence ratio among night and day nurses was compared to a representative measure of parasympathetic nervous system activity by graphically superimposing the HRV coherence ratio with HF power observed during the same time period.

## Results

A total of 30 nurses were recruited, and 18 (60%) completed the study protocol (7 day shift, 11 night shift). The study participants were mostly female (89%), and college educated (100%); and the racial distribution was 44% non-Hispanic European American, and 56% African American or other race (Table 1). The average age (± standard deviation) was 46 ± 10 years. Night nurses were more likely to have > 12 years of lifetime shift work experience relative to day nurses (82% vs. 0%, p < 0.01). Day nurses were more likely to self-report elevated cholesterol levels (86% vs. 18%, p = 0.01) and multivitamin use relative to night nurses (100% vs. 36%, p = 0.01, Table 1).

**Table 1:** Study population demographic and lifestyle characteristics

Characteristic	Total population (n = 18)	Day shift (n = 7)	Night shift (n = 11)	p-value: day versus night
	n (%)	n (%)	n (%)	
Sex				
Female	16 (89)	7 (100)	9 (82)	0.50^b^
Male	2 (11)	0 (0)	2 (18)	
Race				
European American	8 (44)	3 (43)	5 (45)	1.00^b^
African American/other	10 (56)	4 (57)	6 (55)	
Marital status				
Married	14 (78)	5 (71)	9 (82)	1.00^b^
Not married	4 (22)	2 (29)	2 (18)	
Education				
≤ College	9 (50)	3 (43)	6 (55)	1.00^b^
≥ Graduate school	9 (50)	4 (57)	5 (45)	
Body mass index				
Normal or underweight	6 (33)	3 (43)	3 (27)	0.63^b^
Overweight or obese	12 (67)	4 (57)	8 (73)	
Elevated cholesterol				
No	10 (56)	1 (14)	9 (82)	0.01^b^
Yes	8 (44)	6 (86)	2 (18)	
Current smoker				
No	14 (78)	6 (86)	8 (73)	1.00^b^
Yes	4 (22)	1 (14)	3 (27)	
Daily multivitamin use				
No	7 (39)	0 (0)	7 (64)	0.01^b^
Yes	11 (61)	7 (100)	4 (36)	
Work a second job				
No	14 (78)	6 (86)	8 (73)	1.00^b^
Yes	4 (22)	1 (13)	3 (27)	
Lifetime shift work^a^				
≤ 12 years	9 (50)	7 (100)	2 (18)	< 0.01^b^
> 12 years	9 (50)	0 (0)	9 (82)	
Continuous variables (mean ± SD)				
Age (years)	46 (± 10)	46 (± 11)	46 (± 10)	1.00^c^
Smoking (years)	6.0 (± 10)	0.4 (± 1)	9.1 (± 11)	0.14^d^
Alcoholic beverages (days per week)	0.3 (± 0.6)	0.4 (± 0.8)	0.3 (± 0.5)	0.76^d^

^a^12 years was the population median

^b^Fisher’s exact test (2-sided)

^c^Pooled T-test (2-sided)

^d^Wilcoxon exact test (2-sided)

There were no statistically significant differences in the psychometric characteristics of sleep or fatigue, or in psychomotor vigilance, among night nurses relative to those working day shifts, after adjustment for elevated cholesterol and multivitamin use (Table 2). Scores for sleep onset latency among day nurses were moderately elevated relative to night nurses (p = 0.05, Table 2). The self-reported average number of minutes needed for sleep onset among day nurses on work days was 36 ± 24, and was 31 ± 35 among night nurses.

**Table 2:** Average scores for psychometric measures of sleep, fatigue, and performance among permanent day and night shift nurses

Characteristic	Total population (n = 18)	Day shift (n = 7)	Night shift (n = 11)	p-value: day versus night^a^
	Mean (± SD)	Mean (± SD)	Mean (± SD)	
Pittsburg Sleep Quality Index^b^				
Global score	7.1 (± 4.0)	7.1 (± 4.3)	7.0 (± 4.0)	0.40
Sleep duration score	1.5 (± 1.0)	1.5 (± 1.0)	1.5 (± 1.0)	0.94
Sleep latency score	1.4 (± 1.0)	1.6 (± 1.1)	1.2 (± 0.9)	0.05
Daytime dysfunction score	1.0 (± 0.9)	0.4 (± 0.5)	1.4 (± 0.9)	0.63
Sleep disturbance score	1.2 (± 0.4)	1.0 (± 0.0)	1.4 (± 0.5)	0.30
Sleep efficiency score	0.7 (± 1.1)	0.7 (± 1.1)	0.7 (± 1.2)	0.94
Sleep quality score	1.1 (± 0.5)	1.1 (± 0.4)	1.0 (± 0.6)	0.44
Sleep medication score	0.6 (± 1.1)	0.7 (± 1.3)	0.5 (± 1.0)	0.93
Multidimensional fatigue inventory^b^				
General fatigue	11.4 (± 1.7)	11.3 (± 1.3)	11.5 (± 1.9)	0.49
Physical fatigue	11.3 (± 1.2)	11.3 (± 1.8)	11.4 (± 0.8)	0.73
Reduced activity	11.4 (± 1.6)	11.4 (± 1.6)	11.5 (± 1.7)	0.56
Reduced motivation	10.6 (± 2.1)	9.4 (± 1.9)	11.4 (± 2.0)	0.83
Mental fatigue	12.4 (± 0.6)	12.3 (± 0.5)	12.5 (± 0.7)	0.74
Psychomotor vigilance task				
Median reaction time (ms)	278.1 (± 56.7)	269.7 (± 33.2)	283.4 (± 68.7)	0.79
Number of lapses > 500 ms	2.8 (± 5.2)	1.6 (± 1.7)	3.6 (± 6.5)	0.80

*SD* standard deviation

^a^Adjusted for elevated cholesterol and multivitamin use

^b^Higher values represent greater sleep disturbance or more fatigue

**Fig. 1:**
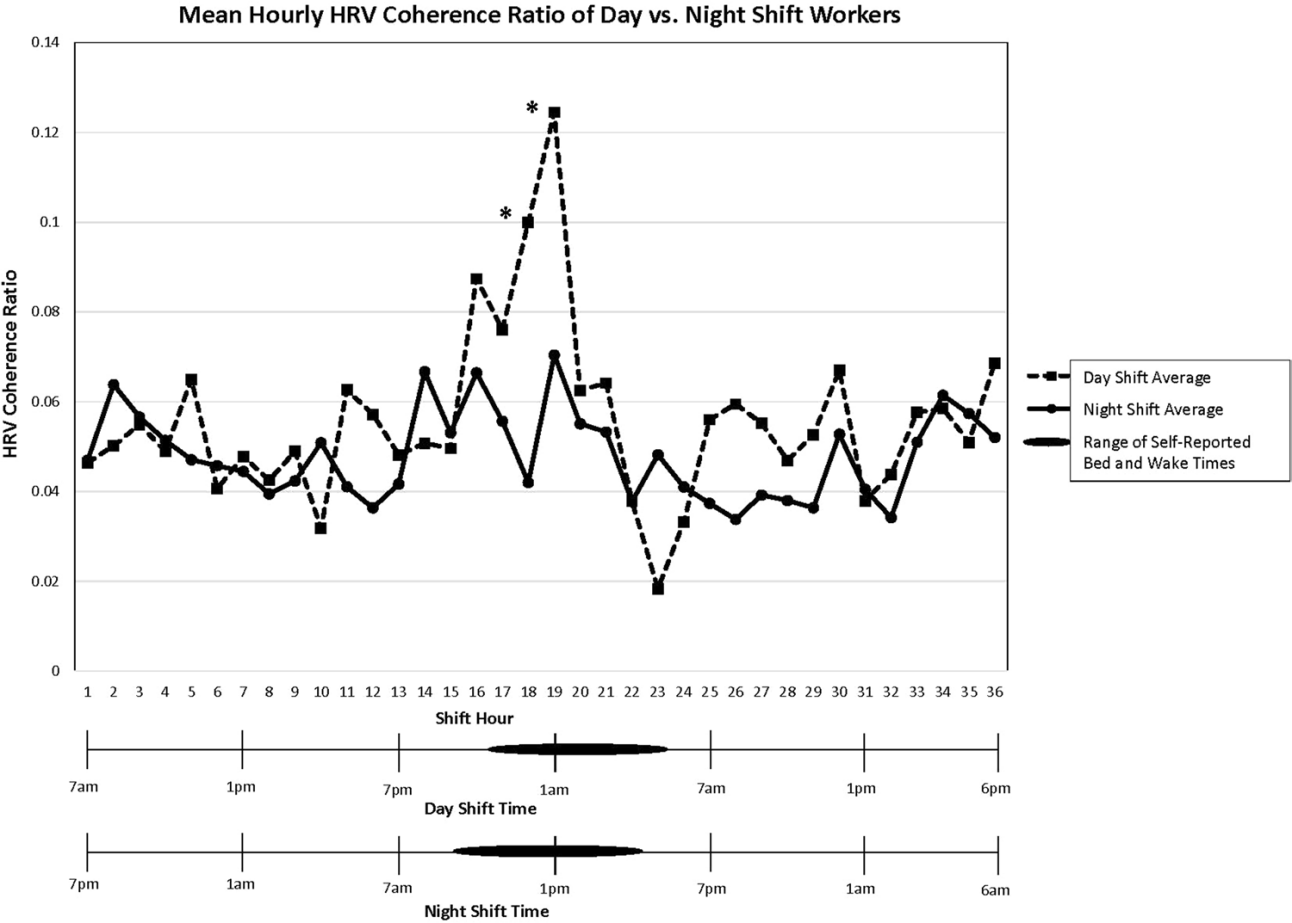
Hourly average HRV coherence ratios among permanent day (n = 7, dashed lines) and night (n = 11, solid lines) shift nurses obtained using FirstBeat ambulatory monitors. Lower axes: clock time and self-reported sleep times for participants on each shift. Day shift average bed time was 10:35 pm, and wake time was 05:15 am. Night shift average bed time: 09:15 am, wake time: 04:04 pm. *p < 0.05 versus night shift average at the same shift hour

**Fig. 2:**
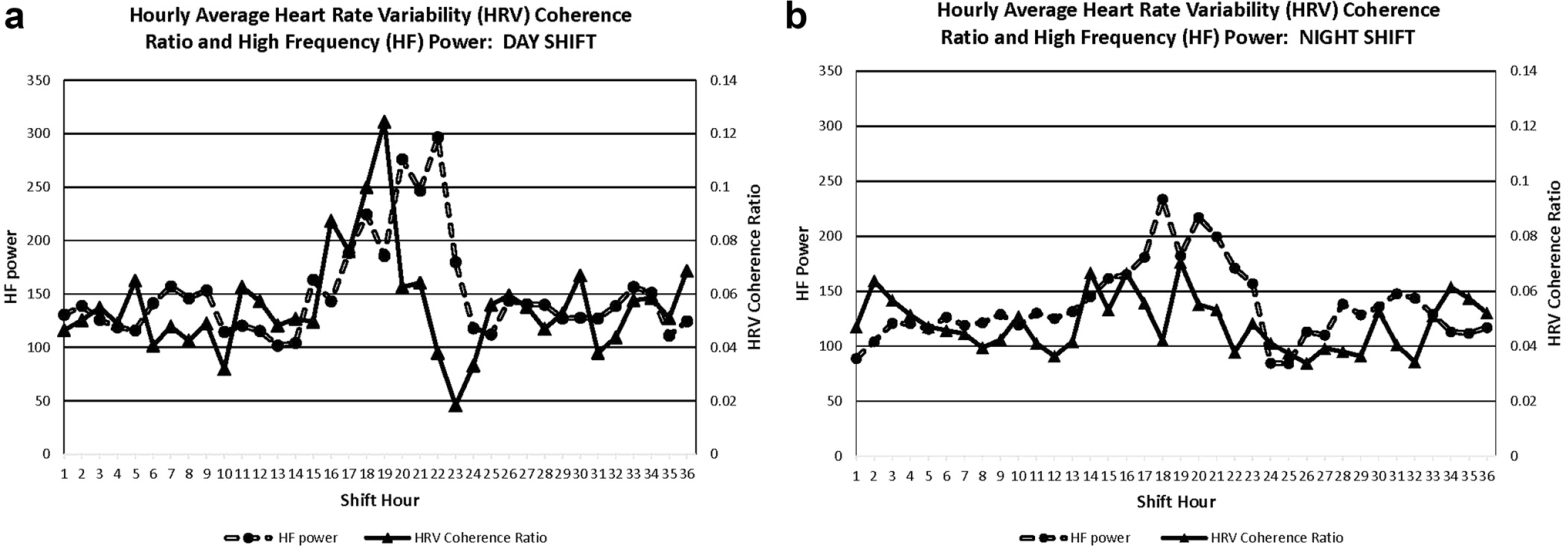
Hourly average HRV coherence ratio and HF power by work shift hour among nurses working permanent day (**a** 7:00 am–7:00 pm, n = 7) or night shifts (**b** 7:00 pm–7:00 am, n = 11)

The range of HRV coherence ratios obtained in this study was consistent with other published values (Berry et al. 2014; Kim et al. 2013). When the hourly average HRV data were organized from the beginning to the end of the 36-h monitoring period (shift hours 1–36, respectively), visual inspection suggested that day nurses had an increase in hourly mean HRV coherence ratios coinciding with their subjective sleep period (shift hours ~ 15–23, Fig. 1), whereas those working permanent nights showed no increase in hourly HRV coherence ratios during their sleep period. Overall, no statistically significant differences in HRV coherence were observed between shifts (F[1,14] = 1.79, p = 0.20), or for the shift by hour interaction (F[35,442] = 1.09, p = 0.34). However, there were statistically significant differences in mean HRV coherence ratios across monitoring hours (F[38,442] = 1.84, p = 0.0023). Before adjustment, statistically significant differences between the day and night shifts were observed at shift hours 18 (day shift: 0.100 ± 0.098, night shift: 0.042 ± 0.024, t[442] = 3.06, p = 0.0024, effect size: 0.81) and 19 (day shift: 0.124 ± 0.129, night shift: 0.070 ± 0.037, t[442] = 3.71, p = 0.0002, effect size: 0.61). The differences at hours 18 and 19 remained statistically significant after adjustment for familywise Type I error (Benjamini–Hochberg adjusted p = 0.0072 and p = 0.0430, respectively, Fig. 1). No other major differences in the hourly means of other HRV parameters were apparent between day and night shift workers during the 36-h study period; for example, there were no statistically significant differences between shifts when comparing hourly averages of HF power, RMSSD, or SDNN (data not shown). However, when data for the HRV coherence ratio and HF power were displayed simultaneously among those working either permanent day (Fig. 2a) or night shifts (Fig. 2b), the pattern of HRV coherence over the 36-h period was similar to the pattern of HF power observed among nurses on the same shift.

## Discussion

To the authors’ knowledge, this study was the first to examine patterns of the HRV coherence ratio over an extended time period in a non-experimental, naturalistic (work/home) setting. Day shift nurses had a clear circadian pattern of HRV coherence, with increases that coincided with initiation of their self-reported sleep period, and subsiding to baseline values by the end of their sleep period. In contrast, no increase in hourly mean HRV coherence ratios was observed among the night nurses during their sleep period. The differences in HRV coherence between shifts suggests an alteration in the pattern of autonomic regulation among the permanent night workers relative to day nurses during their respective sleep periods. However, there were no major differences in self-reported symptoms of sleep, fatigue, or measures of psychomotor vigilance among night workers relative to those working day shifts. The reason for this discrepancy is subject to several possible explanations. Night workers tended to fall asleep sooner than those in the day shift group, which may indicate heightened sleepiness in this group, but also may suggest schedule adaptation among night nurses. In this regard, note that 82% of night nurses were above the median of lifetime shift work for this study population, whereas none of the day nurses had cumulative years of shift work experience exceeding the median. An alternative explanation could be potential information bias (i.e., night nurses may not have been comfortable reporting adverse symptoms of fatigue or sleep). HRV recordings are physiological measures and therefore are not susceptible to this type of bias. However, the absence of differences in PVT measures does not support this explanation. A third possible interpretation is that a diminution in sleep period HRV coherence among the night workers may occur prior to the onset of other symptoms. If that is the case, reductions in sleep period HRV coherence may serve as an early indicator of autonomic dysregulation among night workers, which would be consistent with the role of reduced HRV as a risk indicator in other populations (Tilea et al. 2018; Politano et al. 2008; Tsuji et al. 1996; Zhou et al. 2016; Dekker et al. 2000). The results of this pilot investigation also may have occurred by chance given the sample size. However, patterns of HRV coherence were similar to HF power observed among nurses on the same shift, which tends to corroborate the results obtained using the HRV coherence ratio.

Larger, longitudinal studies are required to confirm whether deficits in HRV coherence are associated with shift work, to determine the timing of onset of such effects after initiating shift work, and what factors may facilitate or prevent their emergence. One approach to exploring these alternatives would be a study that uses the time-honored multi-trait method (Shadish et al. 2002; Campbell and Fiske 1959) to establish a broader array of convergent and discriminant variables that refine and specify the construct of ‘shift work adaptation’. That is, what other ‘shift work adapted’ variables (e.g., biomarkers) might exhibit group differences such as those observed with HRV coherence? Studies of other populations that evaluate: different types of shifts (e.g., timing, rotation, duration, intensity), as well as shift work adaptation strategies (Burch et al. 2009, 2005; Boudreau et al. 2013; Gamble et al. 2011; Arendt 2010), cognitive demands of the workplace, sleep stages, and potentially modifying intrinsic (e.g., chronotype), behavioral, or lifestyle factors are required to help elucidate the potential role of HRV coherence in shift work adaptation.

Optimal cardiovascular health requires a homeostatic balance between sympathetic and parasympathetic ANS activity. Shift work induces stress, enhances sympathetic activity, and is an established risk factor for cardiovascular and metabolic disorders (Reutrakul and Knutson 2015; Faraut et al. 2013; Caruso 2014). Although the pathological mechanism of action is still incompletely understood, changes in HRV may have an important role (Takeda and Maemura 2016; Manfredini et al. 2005; Portaluppi et al. 2012; Aeschbacher et al. 2017; Thayer et al. 2010; Tsuji et al. 1996). Increases in sympathetic activity and reductions in parasympathetic tone have been reported among night workers relative to those working days, or on days off relative to night work, although results have been inconsistent and differences have not always been observed. The importance of measuring HRV among shift workers during their sleep period has recently become more apparent (Chung et al. 2009, 2012; Boudreau et al. 2013; Wehrens et al. 2012; Lindholm et al. 2012), and has been corroborated by studies of HRV following sleep deprivation. For example, a study of 13 healthy male volunteers randomized to five nights of partial sleep deprivation (< 5 h), or five nights of control sleep (> 7 h) found that those with sleep deprivation had statistically significant increases in sympathetic activity (increased LF and decreased HF power) (Dettoni et al. 2012), which was consistent with a prior study that reported increased sympathetic and decreased parasympathetic tone after 36 h of total sleep deprivation (Zhong et al. 2005). Another study found that adaptation to night work was accompanied by lower sympathetic dominance during daytime sleep (Boudreau et al. 2013). Sleep problems are among the most common and persistent symptoms reported among shift workers (Burch et al. 2009; Caruso 2014; Folkard et al. 2005). Results from the current study suggest that the sleep period HRV coherence ratio may be important for distinguishing differences in parasympathetic output among night workers relative to those working days. Statistically significant differences in hourly HRV coherence ratios between day and night workers only occurred during the sleep period, and the differences were consistent with 36-h patterns of HF power, an established indicator of parasympathetic activity, within each shift work group. This finding lends credibility to the use of HRV coherence as an indicator of vagal tone. Another advantage of measuring the HRV coherence ratio is that it likely serves as an indicator of the cardiorespiratory coupling (cardiorespiratory phase synchronization) that occurs during deep sleep and may help facilitate homeostasis between the ANS and cardiopulmonary systems (Tobaldini et al. 2013; Cabiddu et al. 2012; Penzel et al. 2016; Jerath et al. 2014; Garcia et al. 2013). Because HRV coherence can be achieved via behavioral interventions that implement a resonant frequency breathing protocol (i.e., HRV biofeedback) (Lehrer et al. 2000; Garcia et al. 2013; Wheat and Larkin 2010; Lehrer and Gevirtz 2014), the authors speculate that, if the findings of the current study are confirmed, HRV biofeedback may help facilitate shift work adaptation via increases in HRV coherence, which have been associated with improvements in cognitive performance and sleep, as well as reductions in stress, anxiety, and depression (Goessl et al. 2017; Thayer et al. 2009; Lehrer 2018; Lehrer and Gevirtz 2014; Morgan and Mora 2017; Sakakibara et al. 2013), and are all conditions that are commonly encountered among shift workers.

## References

[CR1] Aeschbacher S, Schoen T, Dorig L, Kreuzmann R, Neuhauser C, Schmidt-Trucksass A (2017). Heart rate, heart rate variability and inflammatory biomarkers among young and healthy adults. Annals of Medicine.

[CR2] Amirian I, Andersen LT, Rosenberg J, Gogenur I (2014). Decreased heart rate variability in surgeons during night shifts. Canadian Journal of Surgery.

[CR3] Arendt J (2010). Shift work: coping with the biological clock. Occupational Medicine.

[CR4] Barton J, Spelten E, Totterdell P, Smith L, Folkard S, Costa G (1995). The Standard Shiftwork Index: A battery of questionnaires for assessing shiftwork-related problems. Work and Stress.

[CR5] Basner M, Mollicone D, Dinges DF (2011). Validity and sensitivity of a brief psychomotor vigilance test (PVT-B) to total and partial sleep deprivation. Acta Astronautica.

[CR6] Berry ME, Chapple IT, Ginsberg JP, Gleichauf KJ, Meyer JA, Nagpal ML (2014). Non-pharmacological Intervention for chronic pain in veterans: A pilot study of heart rate variability biofeedback. Global Advances in Health and Medicine: Improving Healthcare Outcomes Worldwide.

[CR7] Boudreau P, Dumont GA, Boivin DB (2013). Circadian adaptation to night shift work influences sleep, performance, mood and the autonomic modulation of the heart. PLoS ONE.

[CR8] Burch JB, Tom J, Zhai Y, Criswell L, Leo E, Ogoussan K (2009). Shiftwork impacts and adaptation among health care workers. Occupational Medicine.

[CR9] Burch JB, Yost MG, Johnson W, Allen E (2005). Melatonin, sleep, and shift work adaptation. Journal of Occupational and Environmental Medicine.

[CR10] Buysse DJ, Reynolds CF, Monk TH, Berman SR, Kupfer DJ (1989). The Pittsburgh Sleep Quality Index: A new instrument for psychiatric practice and research. Psychiatry Research.

[CR11] Cabiddu R, Cerutti S, Viardot G, Werner S, Bianchi AM (2012). Modulation of the sympatho-vagal balance during sleep: Frequency domain study of heart rate variability and respiration. Frontiers in Physiology.

[CR12] Camm AJ, Malik M, Bigger JT, Breithardt G, Cerutti S, Cohen RJ (1996). Heart rate variability—Standards of measurement, physiological interpretation, and clinical use. Circulation.

[CR13] Campbell DT, Fiske DW (1959). Convergent and discriminant validation by the multitrait-multimethod matrix. Psychological Bulletin.

[CR14] Caruso CC (2014). Negative impacts of shiftwork and long work hours. Rehabilitation Nursing.

[CR15] Chung MH, Kuo TB, Hsu N, Chu H, Chou KR, Yang CC (2009). Sleep and autonomic nervous system changes—Enhanced cardiac sympathetic modulations during sleep in permanent night shift nurses. Scandinavian Journal of Work, Environment & Health.

[CR16] Chung MH, Kuo TB, Hsu N, Chu H, Chou KR, Yang CC (2012). Recovery after three-shift work: Relation to sleep-related cardiac neuronal regulation in nurses. Industrial Health.

[CR17] Dekker JM, Crow RS, Folsom AR, Hannan PJ, Liao D, Swenne CA (2000). Low heart rate variability in a 2-minute rhythm strip predicts risk of coronary heart disease and mortality from several causes: The ARIC Study. Atherosclerosis risk in communities. Circulation.

[CR18] Dettoni JL, Consolim-Colombo FM, Drager LF, Rubira MC, Souza SB, Irigoyen MC (2012). Cardiovascular effects of partial sleep deprivation in healthy volunteers. Journal of Applied Physiology.

[CR19] Dinges DF, Pack F, Williams K, Gillen KA, Powell JW, Ott GE (1997). Cumulative sleepiness, mood disturbance, and psychomotor vigilance performance decrements during a week of sleep restricted to 4–5 hours per night. Sleep.

[CR20] Dinges DF, Powell JW (1985). Microcomputer analyses of performance on a portable, simple visual RT task during sustained operations. Behavior Research, Methods, Instruments and Computers.

[CR21] Dunlap ME, Bhardwaj A, Hauptman PJ (2015). Autonomic modulation in heart failure: Ready for prime time?. Current Cardiology Reports.

[CR22] Faraut B, Bayon V, Leger D (2013). Neuroendocrine, immune and oxidative stress in shift workers. Sleep Medicine Reviews.

[CR23] Folkard S, Lombardi DA, Tucker PT (2005). Shiftwork: Safety, sleepiness and sleep. Industrial Health.

[CR24] Gamble KL, Motsinger-Reif AA, Hida A, Borsetti HM, Servick SV, Ciarleglio CM (2011). Shift work in nurses: Contribution of phenotypes and genotypes to adaptation. PLoS ONE.

[CR25] Garcia AJ, Koschnitzky JE, Dashevskiy T, Ramirez JM (2013). Cardiorespiratory coupling in health and disease. Autonomic Neuroscience: Basic & Clinical.

[CR26] Gevirtz R (2013). The promise of heart rate variability biofeedback: Evidence-based applications. Biofeedback.

[CR27] Ginsberg JP, Berry ME, Powell DA (2010). Cardiac coherence and posttraumatic stress disorder in combat veterans. Alternative Therapies in Health and Medicine.

[CR28] Goessl VC, Curtiss JE, Hofmann SG (2017). The effect of heart rate variability biofeedback training on stress and anxiety: A meta-analysis. Psychological Medicine.

[CR29] Hochberg Y (1988). A sharper Bonferroni procedure for multiple tests of significance. Biometrika.

[CR30] IARC (2010). Painting, firefighting, and shiftwork. IARC monographs on the evaluation of carcinogenic risks to humans.

[CR31] Irwin MR, Olmstead R, Carroll JE (2016). Sleep disturbance, sleep duration, and inflammation: A systematic review and meta-analysis of cohort studies and experimental sleep deprivation. Biological Psychiatry.

[CR32] Ito H, Nozaki M, Maruyama T, Kaji Y, Tsuda Y (2001). Shift work modifies the circadian patterns of heart rate variability in nurses. International Journal of Cardiology.

[CR33] Jarczok MN, Jarczok M, Mauss D, Koenig J, Li J, Herr RM (2013). Autonomic nervous system activity and workplace stressors—A systematic review. Neuroscience and Biobehavioral Reviews.

[CR34] Jarvelin-Pasanen S, Ropponen A, Tarvainen MP, Karjalainen PA, Louhevaara V (2013). Differences in heart rate variability of female nurses between and within normal and extended work shifts. Industrial Health.

[CR35] Jensen MA, Garde AH, Kristiansen J, Nabe-Nielsen K, Hansen AM (2016). The effect of the number of consecutive night shifts on diurnal rhythms in cortisol, melatonin and heart rate variability (HRV): A systematic review of field studies. International Archives of Occupational and Environmental Health.

[CR36] Jerath R, Harden K, Crawford M, Barnes VA, Jensen M (2014). Role of cardiorespiratory synchronization and sleep physiology: Effects on membrane potential in the restorative functions of sleep. Sleep Medicine.

[CR37] Khitrov MY, Laxminarayan S, Thorsley D, Ramakrishnan S, Rajaraman S, Wesensten NJ (2014). PC-PVT: A platform for psychomotor vigilance task testing, analysis, and prediction. Behavior Research Methods.

[CR38] Kim S, Zemon V, Cavallo MM, Rath JF, McCraty R, Foley FW (2013). Heart rate variability biofeedback, executive functioning and chronic brain injury. Brain Injury.

[CR39] Lang PJ, Bradley MM (2010). Emotion and the motivational brain. Biological Psychology.

[CR40] Lehrer PM (2018). Heart rate variability biofeedback and other psychophysiological procedures as important elements in psychotherapy. International Journal of Psychophysiology.

[CR41] Lehrer PM, Gevirtz R (2014). Heart rate variability biofeedback: How and why does it work?. Frontiers in Psychology.

[CR42] Lehrer PM, Vaschillo E, Vaschillo B (2000). Resonant frequency biofeedback training to increase cardiac variability: Rationale and manual for training. Applied Psychophysiology and Biofeedback.

[CR43] Lin JM, Brimmer DJ, Maloney EM, Nyarko E, Belue R, Reeves WC (2009). Further validation of the Multidimensional Fatigue Inventory in a US adult population sample. Population Health Metrics.

[CR44] Lindholm H, Sinisalo J, Ahlberg J, Hirvonen A, Hublin C, Partinen M (2012). Attenuation of vagal recovery during sleep and reduction of cortisol/melatonin ratio in late afternoon associate with prolonged daytime sleepiness among media workers with irregular shift work. American Journal of Industrial Medicine.

[CR45] Manfredini R, Boari B, Smolensky MH, Salmi R, la Cecilia O, Malagoni MA (2005). Circadian variation in stroke onset: identical temporal pattern in ischemic and hemorrhagic events. Chronobiology International.

[CR46] McCraty R, Zayas MA (2014). Cardiac coherence, self-regulation, autonomic stability, and psychosocial well-being. Frontiers in Psychology.

[CR47] McDonald JH (2014). Handbook of biological statistics.

[CR48] McMenamin TM (2007). A time to work: Recent trends in shift work and flexible schedules. Monthly Labor Review.

[CR49] Morgan JS, Mora JAM (2017). Effect of heart rate variability biofeedback on sport performance, a systematic review. Applied Psychophysiology and Biofeedback.

[CR50] Park G, Thayer JF (2014). From the heart to the mind: Cardiac vagal tone modulates top-down and bottom-up visual perception and attention to emotional stimuli. Frontiers in Psychology.

[CR51] Penzel T, Kantelhardt JW, Bartsch RP, Riedl M, Kraemer JF, Wessel N (2016). Modulations of heart rate, ECG, and cardio-respiratory coupling observed in polysomnography. Frontiers in Psychology.

[CR52] Politano L, Palladino A, Nigro G, Scutifero M, Cozza V (2008). Usefulness of heart rate variability as a predictor of sudden cardiac death in muscular dystrophies. Acta Myologica.

[CR53] Portaluppi F, Tiseo R, Smolensky MH, Hermida RC, Ayala DE, Fabbian F (2012). Circadian rhythms and cardiovascular health. Sleep Medicine Reviews.

[CR54] Proper KI, van de Langenberg D, Rodenburg W, Vermeulen RCH, van der Beek AJ, van Steeg H (2016). The relationship between shift work and metabolic risk factors: A systematic review of longitudinal studies. American Journal of Preventive Medicine.

[CR55] Puttonen S, Harma M, Hublin C (2010). Shift work and cardiovascular disease—Pathways from circadian stress to morbidity. Scandinavian Journal of Work, Environment & Health.

[CR56] Puttonen S, Viitasalo K, Harma M (2011). Effect of shiftwork on systemic markers of inflammation. Chronobiology International.

[CR57] Reutrakul S, Knutson KL (2015). Consequences of circadian disruption on cardiometabolic health. Sleep Medicine Clinics.

[CR58] Sakakibara M, Hayano J, Oikawa LO, Katsamanis M, Lehrer P (2013). Heart rate variability biofeedback improves cardiorespiratory resting function during sleep. Applied Psychophysiology and Biofeedback.

[CR59] Shadish, W. R., Cook, T. D., & Campbell, D. T. (2002). Construct validity and external validity. In Experimental and quasi-experimental designs for generalized causal inference. Belmont, CA: Wadsworth Cengage Learning.

[CR60] Smets EM, Garssen B, Cull A, de Haes JC (1996). Application of the multidimensional fatigue inventory (MFI-20) in cancer patients receiving radiotherapy. British Journal of Cancer.

[CR61] Smets EM, Grassen B, Bonke B, De Haes J (1995). The Multidimensional Fatigue Inventory (MFI) psychometric qualities of an instrument to assess fatigue. Journal of Psychosomatic Research.

[CR62] Souza BB, Monteze NM, de Oliveira FL, de Oliveira JM, de Freitas Nascimento SN, do Nascimento Neto RM (2015). Lifetime shift work exposure: association with anthropometry, body composition, blood pressure, glucose and heart rate variability. Occupational and Environmental Medicine.

[CR63] Stein PK, Pu Y (2012). Heart rate variability, sleep and sleep disorders. Sleep Medicine Reviews.

[CR64] Takeda N, Maemura K (2016). Circadian clock and the onset of cardiovascular events. Hypertension Research.

[CR65] Thayer JF, Hansen AL, Saus-Rose E, Johnsen BH (2009). Heart rate variability, prefrontal neural function, and cognitive performance: The neurovisceral integration perspective on self-regulation, adaptation, and health. Annals of Behavioral Medicine.

[CR66] Thayer JF, Yamamoto SS, Brosschot JF (2010). The relationship of autonomic imbalance, heart rate variability and cardiovascular disease risk factors. International Journal of Cardiology.

[CR67] Tilea I, Petra D, Ardeleanu E, Hutanu A, Varga A (2018). Clinical conditions and predictive markers of non-dipper profile in hypertensive patients. Acta Medica Marisiensis.

[CR68] Tkachenko O, Dinges DF (2018). Interindividual variability in neurobehavioral response to sleep loss: A comprehensive review. Neuroscience and Biobehavioral Reviews.

[CR69] Tobaldini E, Nobili L, Strada S, Casali KR, Braghiroli A, Montano N (2013). Heart rate variability in normal and pathological sleep. Frontiers in Physiology.

[CR70] Togo F, Takahashi M (2009). Heart rate variability in occupational health—A systematic review. Industrial Health.

[CR71] Toothacker LEE (1993). Multiple comparisons procedures: Quantitative applications in the social sciences.

[CR72] Topolski TD, LoGerfo J, Patrick DL, Williams B, Walwick J, Patrick MB (2006). The rapid assessment of physical activity (RAPA) among older adults. Preventing Chronic Disease.

[CR73] Tsuji H, Larson MG, Venditti FJ, Manders ES, Evans JC, Feldman CL (1996). Impact of reduced heart rate variability on risk for cardiac events. The Framingham Heart Study. Circulation.

[CR74] van Amelsvoort LG, Schouten EG, Maan AC, Swenne CA, Kok FJ (2001). Changes in frequency of premature complexes and heart rate variability related to shift work. Occupational and Environmental Medicine.

[CR75] Videnovic A, Zee PC (2015). Consequences of circadian disruption on neurologic health. Sleep Medicine Clinics.

[CR76] Vogel M, Braungardt T, Meyer W, Schneider W (2012). The effects of shift work on physical and mental health. Journal of Neural Transmission.

[CR77] Wang XS, Armstrong ME, Cairns BJ, Key TJ, Travis RC (2011). Shift work and chronic disease: The epidemiological evidence. Occupational Medicine.

[CR78] Wehrens SM, Hampton SM, Skene DJ (2012). Heart rate variability and endothelial function after sleep deprivation and recovery sleep among male shift and non-shift workers. Scandinavian Journal of Work, Environment & Health.

[CR79] Wheat AL, Larkin KT (2010). Biofeedback of heart rate variability and related physiology: A critical review. Applied Psychophysiology and Biofeedback.

[CR80] Yong LC, Li J, Calvert GM (2017). Sleep-related problems in the US working population: Prevalence and association with shiftwork status. Occupational and Environmental Medicine.

[CR81] Zhong X, Hilton HJ, Gates GJ, Jelic S, Stern Y, Bartels MN (2005). Increased sympathetic and decreased parasympathetic cardiovascular modulation in normal humans with acute sleep deprivation. Journal of Applied Physiology.

[CR82] Zhou X, Ma Z, Zhang L, Zhou S, Wang J, Wang B (2016). Heart rate variability in the prediction of survival in patients with cancer: A systematic review and meta-analysis. Journal of Psychosomatic Research.

